# Venesection as an Anti-Hemorrhagic

**Published:** 1878-11

**Authors:** 


Venesection as an Anti-Hemorrhagic.—Prof. Chauffard is of opinion (*Abeille Medicale*) that at the present time venesection is too much neglected, and especially in cases where it is desirable to open up another channel for blood which tends to become effused at the surface of an organ. He pointed out a case of hæmoptysis at the Necker, occurring in a robust young man who was almost plethoric, a stone-cutter by trade, and therefore of an occupation dangerous to the pulmonary organs. A very abundant hæmoptysis had appeared suddenly three days before his admission, and still continued. Prof. Chauffard having ascertained that no serious lesion existed, ordered venesection to the extent of 500 grammes, and the cure was immediate and complete. This is the reverse of what is met with when a normal hemorrhage which is suppressed gives rise to another in its place. It is not only an accidental hemorrhage which may be thus arrested, but also the hemorrhagic molimen when it becomes dangerous. Thus one of the most important applications of venesection in practice is for the prevention of catamenial congestions persisting after impregnation, and leading to the detachment of the germ—a cause of sterility which is frequently misunderstood. A case in point is that of a lady who, during a period of ten years, repaired to every resort reputed as useful for the prevention of sterility. She was a stout and plethoric person, with very abundant menstruation, which appeared at regular periods. In neither her nor in her husband was there any cause preventive of impregnation. This had probably taken place, but had been rendered invalid by reason of the abundant periodical discharge which had continued. The day before the expected period a small quantity of blood was taken, and then again the next day; and at the end of nine months the lady had her first child, which was followed by others. This cause of sterlility, especially in persons of generous blood, is by no means rare.—*Med. Times and Gazette*, October 12, 1878, from *Presse Belge*, Sept. 29.

